# A Privacy Attack on Multiple Dynamic Match-key based Privacy-Preserving Record Linkage

**DOI:** 10.23889/ijpds.v5i1.1345

**Published:** 2020-08-11

**Authors:** A Vidanage, T Ranbaduge, P Christen, S Randall

**Affiliations:** 1 The Australian National University, Canberra, Australia; 2 Curtin University, Perth, Western Australia, Australia

## Abstract

**Introduction:**

Over the last decade, the demand for linking records about people across databases has increased in various domains. Privacy challenges associated with linking sensitive information led to the development of privacy-preserving record linkage techniques. The multiple dynamic match-key encoding approach recently proposed by Randall et al. (IJPDS, 2019) is such a technique aimed at providing enough privacy for linkage applications while obtaining high linkage quality. However, the use of this encoding in large databases can reveal frequency information that can allow the re-identification of encoded values.

**Objectives:**

We propose a frequency-based attack to evaluate the privacy guarantees of multiple dynamic match-key encoding. We then present two recommendations that can be used in this match-key encoding approach to prevent such a privacy attack.

**Methods:**

The proposed attack analyses the frequency distributions of individual match-keys in order to identify the attributes used for each match-key, where we assume the adversary has access to a plain-text database with similar characteristics as the encoded database. We employ a set of statistical correlation tests to compare the frequency distributions of match-key values between the encoded and plain-text databases. Once the attribute combinations used for match-keys are discovered, we then re-identify encoded sensitive values by utilising a frequency alignment method. Next, we propose two recommendations; one to alter the original frequency distributions and another to make the frequency distributions uniform. Both will help to prevent frequency-based attacks.

**Results:**

We evaluate our privacy attack using two large real-world databases. The results show that in certain situations the attack can successfully re-identify a set of sensitive values encoded using the multiple dynamic match-key encoding approach. On the databases used in our experiments, the attack can re-identify plain-text values with a precision and recall of both up to 98%. Furthermore, we show that our proposed recommendations are able to make this attack harder to perform with only a small reduction in linkage quality.

**Conclusions:**

Our proposed privacy attack demonstrates the weaknesses of multiple match-key encoding that should be taken into consideration when linking databases that contain sensitive personal information. Our proposed recommendations ensure that the multiple dynamic match-key encoding approach can be used securely while retaining high linkage quality.

## Introduction

Privacy-preserving record linkage (PPRL) is the process of linking records that belong to the same individual across different databases while preserving the privacy of the individuals that are represented by the records in these databases [1]. Existing PPRL approaches have a trade-off between linkage quality, scalability, and privacy [1], with some being vulnerable to privacy attacks [2,3,4].

The multiple dynamic match-key approach [5] is a recently proposed method for PPRL which aims to provide privacy protection against frequency attacks, while achieving high linkage quality. As we describe next, the main idea of this approach is to generate distinct hash-codes (called *match-key values*) using attribute value combinations (called *match-keys*) and compare these match-key values across databases to identify matching pairs of records.

### Multiple dynamic match-key approach

We now briefly describe the main steps of multiple dynamic match-key encoding as proposed by Randall et al. [5]. Common notations used throughout the paper are shown in Table 1. We assume a sensitive database, **D**, with *n* records, where each record has *k* attributes. A match-key is a combination of attributes from **D**. Given there are *k* attributes in **D**, there can be 2^*k*^ - 1 attribute combinations. A list of match-keys **mk***_e_* is first selected and then match-key values are created for each record *r* ∈ **D**. These match-key values are hash-codes generated by hashing the concatenated values of each match-key *mk_e_* ∈ **mk***_e_*, as illustrated in Figure 1. An encoded database (consisting of hash-codes) generated from **D** is denoted as **H**, a matrix of hash-codes. Essentially the matrix **H** is generated with *n* rows and *m* columns where *n* = |**D**| and *m* = |**mk**_*e*_|. A row in **H** corresponds to a record *r* ∈ **D** and each row contains a list of *m* hash-codes. A column in **H** corresponds to a certain match-key *mk_e_* ∈ **mk***_e_*, where a given column contains the hash-codes generated for all *r* ∈ **D**. Hence a cell **H**_*ij*_ represents a hash-code, *h_ij_*, generated for the *i*^th^ record using the *j*^th^ match-key, where 1 ≤ *i* ≤ *n* and 1 ≤ *j* ≤ *m*.

**Figure 1:** An example encoding process assuming three attribute combinations (match-keys) where |**mk***_e_*| = 3. The top table refers to the plain-text database attributes before encoding and the bottom table refers to the generated match-key values using HMAC [7]. Note that no match-key value (hash-code) is generated if a match-key has a missing attribute value.

**Plain-text sensitive database (D)**

**Encoded database (H)**

**Table 1: Common notation used throughout the paper.** 

Notation	Definition
D	Sensitive database
V	Plain-text database
*r*	A record in a database
*k*	Number of attribute values in a database
mk*_e_*, mk*_p_*	List of encoded match-keys, and list of plain-text match-keys
H, H_*j_	Encoded matrix of hash-codes, and a column in H which represents a *mk_e_* ∈ mk*_e_*
M, M_*j_	Plain-text match-key matrix, and a column in M which represents a *mk_p_* ∈ mk*_p_*
C, C_i*_, C_*j_	Correlation matrix, a row in C (*mk_p_* ∈ mk*_p_*), and a column in C (correlation metric)
f	A frequency distribution
*i*, *j*	*i*th row and *j*th column in a matrix

The list **mk***_e_* is determined using the probabilistic record linkage method proposed by Fellegi and Sunter [6], which utilises the error characteristics of the databases that are being linked. As described in detail by Randall et al. [5], for each potential match-key a weight score *w* is calculated using the method proposed by Fellegi and Sunter [6]. For the multiple dynamic match-key approach, these weight scores *w* are then compared against a user-defined weight threshold, *w_t_*. Only those match-keys that have a weight score of at least *w_t_* are selected for **mk***_e_* to generate match-key values. The authors of the multiple match-key approach also recommended selecting match-keys with at least two attributes [5].

Due to the large number of match-keys that are possible for *k* attributes (2^*k*^ - (*k*+1) in total), the authors have employed a superset pruning approach to reduce the number of match-keys that are being selected in order to reduce the computational costs [5]. For instance, if the match-key {*FirstName, LastName*} has a total score of at least *w_t_* then all the supersets of this match-key are pruned. This is because having a superset such as {*FirstName, LastName, Gender*} will not result in any additional matches compared to the matches already identified by the match-key {*FirstName, LastName*}.

Once a list of match-keys **mk**_*e*_ is selected, match-key values are generated for each match-key using the corresponding attribute values of each record. To generate match-key values, a keyed cryptographic hash function, such as the hashed message authentication code (HMAC) [7] that uses a hash function and a secret key (known only to the owners of the databases that are being encoded for linkage) can be used. If one of the attribute values used in a match-key is missing, then that match-key value is left empty. Given the list of selected match-keys **mk***_e_*, each record in the database **D** will have up to |**mk**_*e*_| match-key values assigned to it (less if the record has missing values in one or more of the attributes used in **mk**_*e*_). We provide an example of this generation of match-key values in Figure 1. The match-key values for each *r* ∈ **D** are stored as lists (i.e. with an order), and only match-key values at the same position in these lists are compared between records. This will reduce the computational complexity of the comparison since one match-key value of a record will only be compared with one match-key value of another record.

In a linkage protocol, the owners of the sensitive databases to be linked first generate such a list of match-key values for each record in their database. Assuming two databases, **D***_A_* and **D***_B_*, these databases are encoded into two matrices of match-key values (hash-codes), **H***_A_* and **H***_B_*, respectively. In the comparison and classification step, assumed to be conducted by a third party known as the linkage unit [1], the corresponding match-key values (hash-codes in the same column in **H***_A_* and **H***_B_*) are then compared to classify each candidate record pair either as a match or non-match. Following Randall et al. [5], a record pair is classified as a match (assumed to refer to the same individual) if one or more of its match-key values are the same. If none of the corresponding match-key values of a record pair are the same, then the record pair will be classified as a non-match (assumed to refer to different individuals). 

### Contribution

In this paper, we analyse the privacy guarantees of this multiple dynamic match-key approach using a privacy attack that aims to re-identify the plain-text values that were encoded into hash-codes, using a known (publicly available) plain-text database. The attack tries to identify the attributes used for match-keys by analysing the frequency distributions of individual match-key values. Using two large real-world databases, we show that the attack can re-identify plain-text values with an acceptable level of accuracy. We then propose two improvements as countermeasures for multiple match-key encoding and discuss how these methods can strengthen the privacy guarantees of the match-key encoding approach and prevent this type of privacy attacks.

## Methods

We define the following terms which we use to describe the match-key approach and the proposed privacy attack (as also summarised in Table 1). Assume a sensitive database **D** is encoded using the multiple dynamic match-key approach, which results in a matrix **H** of hash-codes, as shown in Figure 1. Each column in **H** contains hash-codes generated using a unique match-key, *mk_e_* ∈ **mk***_e_*. We refer to these columns as *encoded match-keys*. As with existing attacks on PPRL [2,3,4], we assume the adversary has access to a plain-text database **V** with a frequency distribution of attribute values similar to the encoded database **D**. Such plain-text databases can be sourced externally (e.g. a telephone directory or voter database) or internally (if the attack is conducted by an insider who has access to some type of plain-text database that is similar to the encoded database). We then generate a list of match-keys, **mk***_p_*, from the plain-text database **V** to compare against the encoded match-keys. We refer to these match-keys as *plain-text match-keys*. The resulting plain-text match-key matrix is denoted as **M** where each column is a plain-text match-key *mk_p_* ∈ **mk***_p_*. We assume that the plain-text database **V** contains some or all attributes that are used for the encoded match-keys in **D**. We also assume different levels of knowledge of the adversary, as we discuss in the Results section.

As we describe in detail below, the proposed privacy attack consists of five main steps:

1. Calculate the frequency distributions of encoded match-keys (**f***_j_^e^*) and plain-text match-keys (**f***_j_^p^*).
2. Calculate the correlations between the frequency distributions of each encoded match-key H_*j_ ∈ **H** with all plain-text match-keys M_*j_ ∈ **M**, and for each H_*j_ build a correlation matrix, **C**.
3. Select candidate plain-text match-keys for each encoded match-key H_*j_ by comparing correlation values in **C**.
4. Assign highly correlated plain-text match-keys *mk_p_* to each encoded match-key *mk_e_*.
5. Re-identify plain-text values using frequency alignment of plain-text match-keys.

The first two steps of the attack are illustrated in Figure 2. We discuss these two steps in detail in the following two sections.

**Figure 2: An overview of the first two steps of the proposed privacy attack. We first generate the plain-text match-key matrix, M, and then we calculate the frequency distributions, fje and fjp, of all encoded match-keys H*j ∈ H and plain-text match-keys M*j ∈ M. Next, every possible pair of fje and fjp is compared using several correlation metrics. This results in a correlation matrix C for each encoded match-key H*j.**
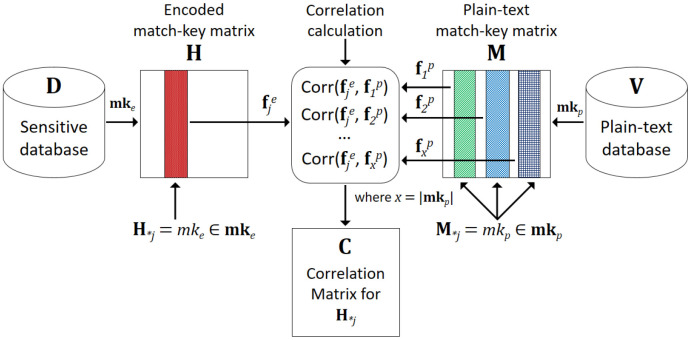


### Step 1: Calculating frequency distributions

In the first step of the attack we calculate the frequency distributions of match-key values, **f***_j_^e^*, for each encoded match-key (i.e. column H_*j_ ∈ **H**) where 1 ≤ *j* ≤ |**mk***_e_*|. Since the match-key values of records are stored as lists (i.e. with an order), we are able to calculate frequency distributions of match-key values for each encoded match-key.

Next, we generate different match-keys of the plain-text database **V**. Assuming there are *k* attributes in **V** (including some or all attributes used for the encoded match-keys) we generate all match-keys from length two to *k*, assuming match-keys have only been generated from combinations of two or more attributes [5]. For the resulting list of plain-text match-keys, **mk***_p_*, for each record *r* ∈ **V**, we generate a list of plain-text match-key values using all *mk_p_* ∈ **mk***_p_*. This results in a matrix of plain-text match-key values, **M**, where a column M_*j_ ∈ **M** represents a plain-text match-key *mk_p_* ∈ **mk***_p_*. Then, for each column M_*j_, we calculate the frequency distribution, **f***_j_^p^*, of concatenated attribute values in *mk_p_* from **M**.

For instance, if there are three attributes *FirstName*, *LastName*, and *BirthYear* in the plain-text match-key database **M**, we can generate four match-keys M_*j_ = {(*FirstName*+*LastName*), (*FirstName*+*BirthYear*), (*LastName*+*BirthYear*), (*FirstName+LastName+BirthYear*)}. Here + is used to represent the string concatenation. We then calculate frequency distributions for all four match-keys. At the end of the first step the adversary has the frequency distributions of all encoded match-keys from **H** (**f***_j_^e^*) and all plain-text match-keys from **M** (**f***_j_^p^*).

### Step 2: Measuring the correlation of frequency distributions

In the second step, we use a series of statistical tests to compare the frequency distributions of all plain-text match-keys, **f***_j_^p^*, with one given encoded match-key, **f***_j_^e^*. For each encoded match-key, H_*j_, we build a correlation matrix, **C** which contains all correlations calculated between each plain-text match-key, M_*j_, and that encoded match-key, H_*j_ (each pair of **f***_j_^e^* and **f***_j_^p^*), using different correlation metrics. Then by comparing these correlation values, we obtain the most similar plain-text match-keys for the current encoded match-key, H_*j_. In the following we discuss this process in more detail.

The statistical tests we use can be divided into two categories. The first consists of basic statistics which can be calculated for a distribution, while the second consists of correlation tests which can be used to compare two distributions. As for the basic statistics we calculate the *mean, standard-deviation, variance*, and *skewness* of two frequency distributions and calculate the absolute difference of those values for different pairs of **f***_j_^e^* and **f***_j_^p^*. Then we employ the following correlation metrics to compare the two distributions **f***_j_^e^* and **f***_j_^p^*:

1. **Earth mover's distance (EMD)** [8]: The EMD is used to measure the dissimilarity between two probability distributions over some feature space. Given one distribution as the baseline, EMD calculates the least amount of work needed to reach the second distribution.
2. **Kolmogorov-Smirnov (KS) test** [9]: The KS test is used to measure the equality of two continuous probability distributions to inspect if one distribution is a sample of another distribution.
3. **Pearson's correlation coefficient** [10]: Given two continuous variables *x* and *y*, Pearson's correlation coefficient measures the correlation between these two variables. The relationship calculation is based on the method of co-variance.
4. **Spearman's rank correlation** [10]: Spearman's Rank correlation measures the ranked correlation between two variables. Specifically, it calculates the strength and direction of association between two ranked variables.
5. **Relative entropy (Kullback-Leibler divergence)** [11]: Relative entropy is used to measure how one probability distribution differs from another reference probability distribution. We use relative entropy to measure how much one frequency distribution is different from the second distribution.
6. **Histogram intersection** [12]: Histogram intersection measures the similarity of two probability distributions by calculating the intersection of their histograms. For this calculation the two histograms of the probability distributions should be of the same length or both these distributions can be placed into a same number of bins using an appropriate binning technique such as equal depth binning. Given two histograms, *x* and *y*, with *b* bins in each, the intersection of the two histograms can be calculated as:
  $$I(x,y)=\sum_{\left(i=1\right)}^{b}min(x_{i},y_{i}),$$
  where *min*() outputs the minimum of *x_i_* and *y_i_*. To normalise the intersection value, we can divide *I(x,y)* by the average of the two histograms:
  $$I{\left(x,y\right)}_{norm}=\frac{2I(x,y)}{\sum_{\left(i=1\right)}^{b}x_{i}+y_{i}}.$$
  In our attack, we use histogram intersection to calculate the intersection of the frequencies of distribution pairs **f***_j_^e^* and **f***_j_^p^*. The higher the value of *I(x,y)_norm_*, the more similar the two distributions are.

These six metrics are commonly used to compare the correlations between probability or frequency distributions. Given we are interested in measuring the correlations between two frequency distributions, we aim to identify correlated match-keys by employing these metrics. Furthermore, our evaluation using these metrics will help us to determine the usability of these correlation metrics in such an attack scenario.

The correlation values calculated using the above statistical tests will be added to a single vector, **c**, of ten values (i.e. four values from the basic statistical metric differences and six values from the above discussed correlation metrics) for each pair of frequency distributions **f***_j_^e^* and **f***_j_^p^*. For each encoded match-key, H_*j_, and its corresponding frequency distribution, **f***_j_^e^*, we obtain a set of correlation vectors by comparing **f***_j_^e^* with the frequency distributions **f***_j_^p^* of all possible plain-text match-keys in **M**. This results in a correlation matrix **C***_j_* (for that particular encoded match-key H_*j_) where **C**_i*_ (row *i*) represents a correlation vector **c** calculated for a certain plain-text match-key and C_*j_ (column *j*) represents a vector of correlations for a certain correlation metric (i.e. one of the above ten tests). In practice it is important to understand which correlation metrics are robust when calculating correlations with different distributions. This aspect is outside the scope of this paper and we considered the performance of all correlation metrics to be equal in our context. In the following steps we describe how we can use the obtained correlation values to filter plain-text match keys that are unlikely to match to encoded match keys.

**Frequency based filtering:** Given there are *k* attributes in **V**, potentially there are 2^*k*^ - (*k*+1) attribute combinations that we need to analyse. Hence, we propose a filtering step to reduce this number of candidate plain-text match-keys by comparing their frequencies. We identify not possible plain-text match-keys through their top most frequent plain-text values. For instance, an encoded match-key with the highest frequency of 50 will not be compared with a plain-text match-key that has a highest frequency of 150. This is assuming that the two databases have a similar number of records and frequency distributions in their attribute values. We define a frequency range parameter, ε, for the range of frequency values that can be used to filter plain-text match-keys. An encoded match-key with highest frequency of *max*(**f***_j_^e^*), will only be compared with a plain-text match-key with highest frequency of *max*(**f***_j_^p^*) if (*max*(**f***_j_^e^*)-ε) ≤ *max*(**f***_j_^p^*) ≤ (*max*(**f***_j_^e^*)+ε). Here *max*() represents the top frequency (highest count) of that particular frequency distribution. The value for ε needs to be adjusted based on the encoded and plain-text database sizes, |**D**| and |**V**|.

### Step 3: Filtering candidate plain-text match-keys

In the third step of the attack we first normalise the correlation values in the matrix **C** for each statistical test using min-max normalisation. Here correlation values are normalised in such a way that the highest correlation will become 1.0 and the lowest correlation will become 0.0. However, the distance values (such as Earth mover's distance and Relative entropy) are normalised in such a way that the highest value will become 0.0 and the lowest value will become 1.0. This ensures that all values obtained using different statistical tests are in the same range and comparable.

Next, for each correlation metric, C_*j_, we sort the plain-text match-keys **mk***_p_* in descending order based on their normalised correlation values. We then select the top plain-text match-keys based on this sorted list. For each pair of plain-text match-keys, *mk_x_* and *mk_x+1_*, we calculate the difference ratio, α*_d_*, of their normalised correlation values. We do this to filter the candidate plain-text match-keys that have the highest correlation values with small differences between them. Only plain-text match-keys that have a difference ratio below a user defined difference ratio limit of α will be considered. Assuming the correlation values of *mk_x_* and *mk_x+1_* are *s_x_* and *s_x+1_* respectively, the difference ratio between those two values, α_*d*_, is calculated as:

$$\alpha_{d}=2\frac{s_{x}-s_{x+1}}{s_{x}+s_{x+1}}.$$

If α_*d*_ ≤ α, then we select both match-keys *mk_x_* and *mk_x+1_*. If α_*d*_ > α then we stop the selection of candidate match-keys at that point. This process is performed for all statistical tests, resulting in a filtered matrix of plain-text match-keys for each encoded match-key. Table 2 shows an example matrix **C** obtained after the filtering process for the encoded match-key (*LastName+Gender+BirthYear*). Plain-text match-keys with crossed-out correlation values are not selected for that particular correlation metric due to having a too low correlation value, as discussed in the above filtering step.

**Table 2: An example correlation matrix C for the encoded match-key (LastName+Gender+BirthYear) after the filtering process discussed in the third step of the attack. Crossed-out values represent the plain-text match-keys that are not selected for the corresponding metric due to their low correlation.** 

Plain-text match-key	Mean	Skewness	…	EMD	Entropy	KS-test
*(FirstName+LastName+Gender)*	0.985	0.541	…	0.967	0.901	0.389
*(FirstName+LastName+BirthYear)*	0.662	0.877	…	0.989	0.578	0.998
*(LastName+Gender+BirthYear)*	0.988	0.921	…	0.969	0.974	0.986

At the end of this step, we obtain one filtered correlation matrix **C***_j_* per encoded match-key, H_*j_. This **C***_j_* will contain plain-text match-keys, *mk_p_*, that have the highest correlation values based on the most similar frequency distributions to that encoded match-key H_*j_.

### Step 4: Re-identifying encoded match-keys

In the fourth step of the attack, we try to identify the correct plain-text match-key for each encoded match-key, H_*j_, from its corresponding matrix **C***_j_*. In this section, since we are analysing a single encoded match-key at a time, we exclude *j* from all notations to improve readability. Therefore, it is important to note that the following process is applied for each encoded match-key H_*j_ separately.

For a given filtered matrix **C**, we first calculate an overall correlation, *a_c_*, of each plain-text match-key *mk_p_* by averaging the correlation value vector of that match-key. As we discussed above, this vector consists of the normalised correlation values for the ten different statistical tests. Given that a plain-text match-key *mk_p_* ∈ **C** has a correlation vector **c**_*p*_, the overall correlation *a_c_* can be calculated as:

$$a_{c}=\frac{\sum_{\left(i=1\right)}^{10}c_{i}\in c_{p}}{10}.$$

Next we look at the number of unique match-key values the plain-text match-key (number of unique concatenated plain-text values) and the encoded match-key (number of unique hash-codes) have. Note that even if the distributions of an encoded match-key and a plain-text match-key is similar these two match-keys might have different numbers of unique values. Therefore, apart from correlation measurements, the number of unique values can also be used to compare the two match-keys. If the encoded match-key has *q_e_* unique values and a plain-text match-key *mk_p_* ∈ **C** has *q_p_* unique values, we calculate the difference ratio *d_c_* of those values as:

$$d_{c}=2\frac{abs(q_{p}-q_{e})}{q_{p}+q_{e}},$$

where *abs*() calculates the absolute value. We next calculate the average of the two values, *a_c_* and *d_c_*. This can be calculated as a weighted average, where we assign certain weights to each value. Assuming the weight values for *a_c_* and *d_c_* are ω and 1 - ω respectively (with 0 ≤ ω ≤ 1), the average value *s_c_* is calculated as:

$$s_{c}=\omega a_{c}+(1-\omega )d_{c}.$$

For each encoded match-key we obtain |**C**| of these average values where |**C**| is the number of plain-text match-keys assigned to that encoded match-key. We then sort the plain-text match-keys according to these *s_c_* values and assign the plain-text match-key with the highest *s_c_* value to that encoded match-key. We repeat this step for all the encoded match-keys H_*j_. At the end of this step we get a ranked list of plain-text match-keys that are likely to be more similar to each of the encoded match-keys.

Based on the superset pruning method suggested by the authors of the multiple dynamic match-key approach [5], we can also check for super- or subsets in the identified plain-text match-keys for each encoded match-key H_*j_. If any of the encoded match-keys, H_*j_, has already been assigned to a plain-text match-key *mk_p_^x^*, then any of the super- or subsets of the *mk_p_^x^* cannot be assigned to another encoded match-key, under the assumption that the first assignment of *mk_p_^x^* is correct. An example is discussed in the Introduction section.

### Step 5: Re-identification of plain-text values

Once the plain-text match-keys for each encoded match-key H_*j_ are identified, the final step of the attack is to perform plain-text value re-identification by applying a frequency alignment method [2]. For each encoded match-key, H_*j_, we obtain the frequency distribution of match-key values, and for the identified plain-text match-key, *mk_p_^j^*, of H_*j_, we obtain the frequency distribution of the corresponding plain-text values. Both these frequency distributions are then sorted in descending order as shown in Table 3. First, we adjust these frequency values according to the sizes of the databases **D** and **V**. We can adjust either the frequency values of match-keys by multiplying each value by |**V**| / |**D**|, or alternatively we can adjust the frequency values of plain-text values by multiplying each value by |**D**| / |**V**|. Then we loop over each pair of frequency aligned match-key value and plain-text value and assign the *i*^th^ plain-text value to the corresponding *i*^th^ match-key value. We continue this process until the differences of their frequencies is greater than Δ*_t_*, a user defined threshold. Given the frequencies of the *i*^th^ match-key value and plain-text values are *f_j_^i^* and *f_c_^i^* we calculate the frequency difference Δ*_i_* as:

$$\Delta_{i}=2\frac{abs(f_{j}^{i}-f_{c}^{i})}{f_{j}^{i}+f_{c}^{i}},$$

**Table 3: An example of plain-text alignment for match-key FirstName+LastName+BirthYear (F+L+B) using frequency values of match-key values and plain-texts values. As discussed in step 5 of the attack, record d1 from the encoded database D will be aligned with record v1 from the plain-text database V. Since all three records d2, d3, and d4 have a frequency of 7 and the three records v2, v3, and v4 in the plain-text database also have the same frequency, we assign each of d2, d3, and d4 to all three values of v2, v3, and v4. Similarly, we assign each of d5 and d6 to both values of v5 and v6 since they all have a frequency of 4.** 

Encoded database D			Plain-text database V		
	
Record ID	Match-key value	Frequency	Record ID	(F+L+B)	Frequency
d1	heYcgrjawf3AVtt	10	v1	brittany+nicole+1987	10
d2	JbsJvZQ7lucFDcE	7	v2	brian+johnson+1968	7
d3	NDZ5v3pzT7tvlko	7	v3	james+smith+1991	7
d4	1XW13iYzExn4KGZ	7	v4	ronald+young+1982	7
d5	5qIiMWET4suKARu	4	v5	ashley+johnson+1975	4
d6	b97VABaDFZSg9OM	4	v6	johnny+motley+1989	4

where *abs*() returns the absolute value. If Δ*_i_* > Δ*_t_*, then we stop the alignment of plain-text values to encoded match-key values (hash-codes).

Furthermore, if several adjacent frequencies of match-key values and plain-text values in the sorted lists are the same then it will be difficult to determine which plain-text value corresponds to which match-key value. For instance if *f_j_^i^* = *f_j_*^*i*+1^ = *f_c_^i^* = *f_c_*^*i*+1^, we cannot be certain that the *i*^th^ match-key value refers to *i*^th^ plain-text or (*i*+1)^th^ plain-text value. In such scenarios we assign both the *i*^th^ and (*i*+1)^th^ plain-text values to both the *i*^th^ and (*i*+1)^th^ match-key values, respectively. An example of this plain-text alignment process is illustrated in Table 3. As shown, records with the same frequency values are aligned together in order to improve the accuracy of the alignment. It is also important to note that this alignment works when the underlying attribute value distributions are similar in both **V** and **D**. In cases where attribute value distributions are dissimilar this frequency alignment can potentially lead to incorrect assignments.

Using the above discussed process we can now identify the combined plain-text values from **V** for the most frequent match-key values in each encoded match-key H_*j_. In the following section we describe experiments conducted using our proposed attack method and discuss the results we obtained.

## Results

We evaluated our proposed attack method on the multiple dynamic match-key approach [5] using two large real-world databases. We investigated different parameter settings under different attack scenarios to measure the feasibility of our attack in real linkage situations.

### Databases

We first used a North Carolina Voter Registration (NCVR) database Available at: http://dl.ncsbe.gov/?prefix=data, where we used six snapshots collected in October, August, and February 2019, October 2018 and 2017, and October 2011. We used the snapshot from October 2019 as the sensitive encoded database, **D**, and the other snapshots as the plain-text databases, **V**, to represent several time intervals (2 months, 8 months, 1 year, 2 years, and 8 years, respectively) between the encoded and the plain-text databases. Our aim is to evaluate how the temporal differences in records, such as attribute changes, between databases affects the accuracy and scalability of the attack. Our hypothesis is that the larger the time difference the larger the differences in attribute value distributions between **D** and **V**, as more voters change their names and/or addresses.

The second database was the Michigan voter registration (MVR) databaseAvailable at: http://michiganvoters.info, where we used four snapshots collected in September and January 2016, September 2014, and June 2013. We used the snapshot from September 2016 as the sensitive encoded database, **D**, and the other snapshots as the plain-text databases, **V**. As with the NCVR databases, our aim is to evaluate how the time difference between databases affects the attack.

The selected databases contained between 6,233,661 and 8,114,702 records. The number of changes in attribute values monotonically increase as the time differences between database pairs increases for both the NCVR and MVR databases. For example, between the October 2019 and October 2011 snapshots of NCVR, the attributes *StreetAddress* and *MiddleName* had 47.7% and 8.1% changes in their values, respectively, while between the September 2016 and June 2013 snapshots of MVR, the attributes *StreetNumber* and *LastName* had only 2% and 1% changes in their values, respectively.

### Evaluation criteria

To evaluate the quality of the identification of plain-text match-keys for each encoded match-key H_*j_, we analysed the top three to five plain-text match-keys assigned for each encoded match-key and see whether the attack was able to identify the correct match-key with a high correlation. We then evaluated the accuracy of the re-identified plain-text values for match-key values using precision and recall [13,14] of how many plain-text values identified are correct and how many correct values are in the identified set of plain-text values.

We also define three different attack scenarios based on the assumed knowledge an adversary has about the match-key encoding settings. The scenarios we considered are as follows;

- **Knowledge on attribute combinations (Comb)**: In this scenario we assume that the adversary has knowledge about the actual attribute combinations (match-keys) used in the match-key generation process, but does not know which encoded match-key H_*j_ corresponds to which plain-text match-key M_*j_. In this situation the adversary would not have to consider all possible plain-text match-keys. Only the known combinations need to be evaluated to measure the correlation of frequency distributions. This scenario can be considered as the best possible attack scenario on an encoded database from an adversary’s point of view.
- **Knowledge on used attributes (Attr)**: In this scenario we assumed that the adversary has knowledge only about the set of attributes used for the match-key generation, but does not know which combinations of attributes are used. Unlike the previous scenario, in this situation the adversary would have to conduct the first step of the attack (as discussed in the Methods section) using all possible plain-text match-keys to identify the best matching plain-text match-keys for each encoded match-key, and then perform the second step of the attack.
- **Domain knowledge (Dom)**: In this final scenario we assumed that the adversary does not have any specific knowledge about the attributes, nor the match-keys, used in the match-key generation process. The adversary only knows what kind of entities are encoded in **H** (such people with their names, addresses, and so on) and therefore can partially assume what kind of quasi-identifiers are being used for the encoding. Hence, the adversary needs to run the attack on all possible match-keys using all attributes available in **V** that potentially were encoded into match-keys. This scenario can be considered as the worst case scenario on an encoded database for an adversary.

We implemented all attack methods using Python 2.7 and the experiments were run on a server with 64-bit Xeon 2.1 GHz 16-Core CPU, 512 GBytes of memory and running Ubuntu 18.04. To facilitate reproducibility the prototype programs are available from: https://dmm.anu.edu.au/pprlattack/.

For attack parameter settings, we set α = 0.05, ω = 0.7, Δ_*t*_ = 0.2. An adaptive value is selected for the parameter ε based on the maximum frequency value of the considered encoded match-key. A ratio from 0.1 to 0.9 was first selected and then the selected ratio is multiplied by the maximum frequency value of the encoded match-key to calculate ε. These settings provided good results in a series of set-up experiments.

The parameter α can be set to an appropriate value by analysing the correlation values obtained for each correlation metric. If the frequency distributions of the encoded and plain-text match-keys are similar then α can be set to a low value such as α < 0.1. Inversely, with contrasting frequency distributions, α can be set to a higher value such as α = 0.3 to select a wide range of candidate plain-text match-keys. However, setting α to even a higher value such as α > 0.4 will potentially result in a large selection of candidate plain-text match keys. We set the weight ω of to a higher value than 0.5 because the average correlation value *a_c_* was consistently more accurate in correctly identifying encoded match-keys than the difference ratio *d_c_* in our experiments. The value for Δ_*t*_ should be set to a low value such as Δ_*t*_ < 0.3 to get accurate re-identifications. If the frequency distributions of the two databases are known to have differences, a higher value such as Δ_*t*_ = 0.4 can be set. This should however be done carefully because this could lead to inaccurate plain-text re-identifications, because allowing substantial differences in value distributions to be mapped can be wrongly used by the attack to map dissimilar plain-texts with each other. It is also worth noting that the optimal values for these parameters are data dependent and therefore should be set over several iterations of experiments.

### Match-key generation

Following the originally proposed dynamic match-key encoding [5], we used the following sets of attributes for the experiments for the two databases NVCR and MVR.

- NCVR: *FirstName, MiddleName, LastName, BirthYear, StreetAddress*, and *ZipCode*
- MVR: *FirstName, MiddleName, LastName, BirthYear, StreetNumber, StreetName,* and *ZipCode*

These attributes are commonly used in linking records across different databases [1]. Following Randall et al. [5], we selected a list of match-keys that provided the highest linkage quality (according to the F-measure) using the above attributes to encode the sensitive databases.

## Discussion

In Tables 4 and 5 we show the re-identification results for each encoded match-key used in different databases from different time intervals using the NCVR and MVR databases. As can be seen, with NCVR we are able to identify the attribute combinations used for each encoded match-key with high accuracy despite the time difference and corresponding percentages of attribute changes between these databases. The correct match-key was always amongst the top three identified plain-text match-keys. Furthermore, the attack was able to identify the correct combination as the first (the match-key with highest correlation) 20 out of 24 times.

**Table 4: Re-identification results of encoded match-keys for the NCVR databases with different time intervals. The attributes used are FirstName (F), MiddleName (M), LastName (L), BirthYear (B), StreetAddress (S), and ZipCode (Z). We show the top three plain-text match-keys identified for each encoded match-key and the sc value of each plain-text match-key when compared with a corresponding encoded match-key. In the left-most column we also show the maximum frequency value of the frequency distribution of that match-key. The value None shows where the particular match-key is not used for the encoding of the corresponding database due to not having an overall score above wt, as described in the Introduction section.** 

Encoded match-key with its top frequency	Identified plain-text match-keys with different time intervals D to V			

	2 months	8 months	2 years	8 years
F+M+L+B+S 4	F+M+L+B+S / 0.996			
	M+L+B+S+Z / 0.895	*None*	*None*	*None*
	F+M+B+S+Z / 0.893			
F+L+B+S+Z 4	F+L+B+S+Z / 0.983	F+L+B+S+Z / 0.964		
	F+M+L+B+Z / 0.917	F+M+L+B+Z / 0.909	*None*	*None*
	F+M+L+B+S / 0.908	F+M+L+B+S / 0.906		
F+M+L+B+Z 4	F+M+L+B+Z / 0.981		
	F+L+B+S+Z / 0.948	*None*	*None*	*None*
	M+L+B+Z / 0.922			
F+M+L+S+Z 4	F+M+L+S+Z / 0.990	F+M+L+S+Z / 0.989		
	F+L+S+Z / 0.912	F+L+S+Z / 0.905	*None*	*None*
	F+M+S+Z / 0.776	F+M+S+Z / 0.763		
M+L+B+S+Z 4	M+L+B+S+Z / 0.982	M+L+B+S+Z / 0.975	M+L+B+S+Z / 0.967	
	F+M+B+S+Z / 0.950	F+M+B+S+Z / 0.963	F+L+B+S+Z / 0.915	*None*
	F+M+L+B+S / 0.893	F+M+L+B+S / 0.867	F+M+L+B+S / 0.912	
F+M+B+S+Z 5	F+M+B+S+Z / 0.997	F+M+B+S+Z / 0.973	M+L+B+S+Z / 0.966	
	M+L+B+S+Z / 0.964	M+L+B+S+Z / 0.971	F+M+B+S+Z / 0.962	*None*
	F+M+L+B+S / 0.915	F+M+L+B+S / 0.922	F+M+L+B+S / 0.936	
F+M+L+B 8		F+M+L+B / 0.991	F+M+L+B / 0.981	F+M+L+B / 0.925
	*None*	F+M+L+S / 0.780	M+L+S+Z / 0.781	F+M+L+Z / 0.731
		M+L+S+Z / 0.774	F+M+L+Z / 0.776	M+L+B+Z / 0.710
F+M+L+Z 6			F+M+L+Z / 0.986	F+M+L+Z / 0.955
	*None*	*None*	M+L+B+Z / 0.973	M+L+B+Z / 0.936
			F+L+B+Z / 0.916	F+L+B+Z / 0.843
F+L+B+Z 4			F+L+B+Z / 0.984	M+L+B+Z / 0.943
	*None*	*None*	F+L+B+S+Z / 0.957	F+M+L+Z / 0.874
			M+L+B+Z / 0.955	F+L+B+Z / 0.868
F+M+L+S 20			F+M+L+S / 0.982	
	*None*	*None*	F+B+S+Z / 0.721	*None*
			M+B+S+Z / 0.639
F+L+S+Z 4			F+L+S+Z / 0.987	
	*None*	*None*	F+M+L+S+Z / 0.940	*None*
			F+M+S+Z / 0.811	
L+B+S+Z 14				L+B+S / 0.938
	*None*	*None*	*None*	L+B+S+Z / 0.928
				F+M+S+Z / 0.920
M+B+S+Z 26				M+B+S+Z / 0.965
	*None*	*None*	*None*	M+B+S / 0.960
				F+B+S+Z / 0.874
F+B+S+Z 11				F+B+S / 0.916
	*None*	*None*	*None*	F+B+S+Z / 0.915
				F+M+B+Z / 0.795

**Table 5: Re-identification results of encoded match-keys for the MVR databases with different time intervals. The attributes used are FirstName (F), MiddleName (M), LastName (L), BirthYear (B), StreetNumber (Sd), StreetName (Sn), and ZipCode (Z). We show the top five plain-text match-keys identified for each encoded match-key and the sc value of each plain-text match-key.** 

Encoded match-key with its top frequency	Identified plain-text match-keys with different time intervals D to V		
	8 months	2 years	3 years
L+F+M+B+S_n_	L+F+M+B+S_d_+S_n_ / 0.830	L+F+M+B+S_d_+S_n_ / 0.844	L+F+M+B+S_d_+S_n_ / 0.844
	L+F+M+B+S_d_+Z / 0.827	L+F+M+B+S_d_+Z / 0.839	L+F+M+B+S_d_+Z / 0.840
	L+F+M+B+S_n_+Z / 0.827	L+F+M+B+S_n_+Z / 0.838	L+F+M+B+S_n_+Z / 0.837
	L+F+M+B+S_d_ / 0.825	L+F+M+B+S_d_ / 0.835	L+F+M+B+S_d_ / 0.835
	L+F+M+B+S_n_ / 0.825	L+F+M+B+S_n_ / 0.834	L+F+M+B+S_n_ / 0.833
L+F+M+B+S_d_	L+F+M+B+S_d_+S_n_ / 0.830	L+F+M+B+S_d_+S_n_ / 0.844	L+F+M+B+S_d_+S_n_ / 0.844
	L+F+M+B+S_d_+Z / 0.827	L+F+M+B+S_d_+Z / 0.839	L+F+M+B+S_d_+Z / 0.840
	L+F+M+B+S_n_+Z / 0.827	L+F+M+B+S_n_+Z / 0.838	L+F+M+B+S_n_+Z / 0.837
	L+F+M+B+S_d_ / 0.825	L+F+M+B+S_d_ / 0.835	L+F+M+B+S_d_ / 0.835
	L+F+M+B+S_n_ / 0.825	L+F+M+B+S_n_ / 0.834	L+F+M+B+S_n_ / 0.833
L+M+B+S_d_	L+M+B+S_n_+Z / 0.974	L+M+B+S_d_+S_n_+Z / 0.979	L+M+B+S_d_+Z / 0.920
	L+F+M+B+S_d_+Z / 0.960	L+M+B+S_d_+Z / 0.952	L+F+B+S_n_+Z / 0.910
	L+F+M+B+S_d_ / 0.954	L+M+B+S_d_ / 0.938	L+M+B+S_n_+Z / 0.890
	L+F+M+B+S_n_ / 0.953	L+M+B+S_d_+S_n_ / 0.911	L+F+B+S_n_ / 0.889
	L+M+B+S_d_ / 0.946	L+F+B+S_n_+Z / 0.894	L+M+B+S_d_+S_n_ / 0.884
F+M+B+S_d_	L+M+B+S_n_ / 0.970	L+M+B+S_n_ / 0.967	F+M+B+S_d_+S_n_+Z / 0.957
	F+M+B+S_d_+Z / 0.947	F+M+B+S_d_+S_n_ / 0.965	F+M+B+S_d_+S_n_ / 0.936
	L+F+M+S_d_+S_n_ / 0.929	F+M+B+S_d_+S_n_+Z / 0.953	L+F+M+S_d_+S_n_ / 0.934
	F+M+B+S_d_+S_n_ / 0.928	F+M+B+S_d_+Z / 0.949	L+F+M+S_d_+S_n_+Z / 0.927
	L+F+M+S_d_+Z / 0.918	L+M+B+S_n_+Z / 0.947	F+M+B+S_d_+Z / 0.926
L+F+B+S_d_	L+F+B+S_d_+Z / 0.957	L+F+M+B+Z / 0.953	L+F+B+S_d_ / 0.953
	L+F+B+S_d_+S_n_ / 0.955	L+F+B+S_d_+Z / 0.947	L+F+B+S_d_+Z / 0.950
	L+F+M+B+S_n_ / 0.933	L+F+B+S_d_+S_n_+Z / 0.945	L+F+B+S_n_+Z / 0.908
	L+F+M+B+S_d_ / 0.933	L+F+B+S_d_+S_n_ / 0.900	L+F+B+S_d_+S_n_+Z / 0.903
	L+F+M+B+S_d_+S_n_ / 0.922	L+M+B+S_d_+Z / 0.894	L+F+B+S_d_+S_n_ / 0.883
L+F+M+S_d_	L+F+M+S_d_ / 0.978	L+F+M+S_d_ / 0.987	L+F+M+S_d_+S_n_ / 0.985
	L+M+B+S_n_ / 0.976	L+F+M+S_d_+S_n_+Z / 0.987	L+F+M+S_d_+S_n_+Z / 0.977
	L+F+M+S_d_+Z / 0.974	L+F+M+S_d_+Z / 0.985	F+M+B+S_d_+S_n_ / 0.974
	L+F+M+S_d_+S_n_+Z / 0.965	L+F+M+S_d_+S_n_ / 0.984	L+F+M+S_d_+Z / 0.973
	L+F+M+S_d_+S_n_ / 0.962	F+M+B+S_d_+S_n_+Z / 0.975	L+F+M+S_d_ / 0.972
L+F+M+B+S_d_	L+F+M+B+S_d_+S_n_ / 0.830	L+F+M+B+S_d_+S_n_ / 0.844	L+F+M+B+S_d_+S_n_ / 0.844
	L+F+M+B+S_d_+Z / 0.827	L+F+M+B+S_d_+Z / 0.839	L+F+M+B+S_d_+Z / 0.840
	L+F+M+B+S_n_+Z / 0.827	L+F+M+B+S_n_+Z / 0.838	L+F+M+B+S_n_+Z / 0.837
	L+F+M+B+S_d_ / 0.825	L+F+M+B+S_d_ / 0.835	L+F+M+B+S_d_ / 0.835
	L+F+M+B+S_n_ / 0.825	L+F+M+B+S_n_ / 0.834	L+F+M+B+S_n_ / 0.833

With the MVR databases the re-identification accuracy of the match-keys are lower compared to the NCVR databases. However, as can be seen in Table 5, the identified match-keys are always a superset or a subset of the correct match-key. The correct match-key was amongst the top five identified match-keys 17 out of 21 re-identifications. The lower accuracy results are caused by having low and similar values in the frequency distributions **f***_j_^e^* of match-keys. As shown in Table 5, the highest frequency values of encoded match-keys in MVR are only 3 and 2 compared to the NCVR databases where they are ranging from 4 to 26. Therefore, the correlation values measured using different plain-text match-keys (especially the supersets and subsets of the correct match-key) are all highly similar and it is difficult to distinguish the correct match-key from other possible candidates. While the results on the NCVR databases illustrate the robustness of the attack in re-identifying encoded match-keys when there is enough frequency information in match-keys, at the same time the results on the MVR databases highlight the need of enough frequency information and distinct frequency distributions for the attack to be highly successful.

Table 6 shows the precision, recall, and the number of re-identifications of plain-text values for the NCVR and MVR databases. These are the results obtained in the final step of the attack as discussed in the Methods section. As can be seen, with the NCVR databases, the attack was able to re-identify plain-text values encoded in match-key values with high accuracy when the time difference between databases was small. However, both precision and recall drop when the time difference between databases is increasing. This is because, even if the attack was able to identify the correct attribute combinations used for encoded match-keys by analysing the frequency distributions, exact re-identifications of plain-text values as described in the fifth step of the attack are difficult due to having changes in actual attribute values. With different scenarios the attack maintains fairly consistent results. As expected with the **Dom** scenario the numbers of re-identifications are slightly lower than the other scenarios because the accuracy of the re-identification of match-keys is lower.

**Table 6: Precision (Prec) and recall (Reca) results for plain-text re-identifications for most frequent match-key values. The total number of re-identifications (Total re-ident.) identified by each experiment is also presented. Results are shown for both the NCVR and MVR databases and for different time durations in D and V. Results are further categorised into attack scenarios Comb, Attr, and Dom.** 

	Time interval D to V	Comb		Attr		Dom	
		
		Prec / Reca (%)	Total re-ident.	Prec / Reca (%)	Total re-ident.	Prec / Reca (%)	Total re-ident.
NCVR	2 months	98.6 / 98.4	1137	98.6 / 98.4	1137	91.8 / 91.1	951
	8 months	81.7 / 81.4	14415	81.7 / 81.4	14415	77.0 / 80.9	736
	1 year	69.7 / 69.3	705	58.0 / 59.9	705	70.4 / 70.1	887
	2 years	47.7 / 41.4	451	58.3 / 54.0	1481	61.4 / 62.1	1480
	8 years	30.0 / 30.4	1196	31.7 / 29.4	1186	16.3 / 15.3	1798
MVR	8 months	48.6 / 48.5	4972	46.6 / 35.6	45	14.2 / 11.3	30
	2 years	32.1 / 33.2	1675	37.2 / 37.7	1657	42.4 / 45.6	1657
	3 years	26.6 / 27.6	1671	32.3 / 32.3	1727	28.7 / 32.0	1735

With the MVR databases, the plain-text alignment accuracy is relatively low compared to the NCVR database experiments. This is because plain-text alignment is done only using the top most selected plain-text match-key as discussed in the Methods section. According to Table 5, the attack was not able to identify the correct encoded match-key as the top selected plain-text match-key most of the times. Therefore, the plain-text alignment did not perform as accurately as with NCVR databases. However, we are still able to identify some of the attribute values correctly because all identified plain-text match-keys for the MVR are either a superset or a subset of the correct encoded match-keys.

With regard to the time efficiency, the first and the second steps are the most time consuming steps of the attack requiring a maximum of 3,840 and 7,352 seconds respectively. The third and fourth steps took less than one second for all experiments, and the fifth step took a maximum of 307 seconds. Overall the attack took less than 3.5 hours to complete for all databases and attack scenarios Additional evaluation results of the proposed attack such as time complexity and database analysis (excluded from the paper due to space constraints) are available at: https://dmm.anu.edu.au/pprlattack/.

Out of all the tests we utilised to compare frequency distributions, the correlation metrics *Earth mover’s distance, KS test, Pearson's correlation, Spearman’s rank correlation, Relative entropy*, and *Histogram intersection* provided consistently good results with different attack scenarios and databases with different time intervals compared to the four basic statistical measures used. This is due to the ability of correlation metrics to identify common characteristics of distributions even with differences in actual frequency values.

### Privacy improvements and recommendations

Considering the identified privacy vulnerabilities in the match-key generation process, we now propose two recommendations to strengthen the privacy guarantees of the multiple dynamic match-key encoding approach. We further show empirical evidence to support our claim of using these recommendations to make the match-key encoding resilient to frequency-based privacy attacks.

**Recommendation 1:** As we discussed in the Introduction section, match-keys are encoded and stored as lists so that only corresponding match-key values are compared when matching two records. For instance, if records are encoded using four match-key values, [*mk_e_^1^, mk_e_^2^, mk_e_^3^, mk_e_^4^*], when a record pair r1 and r2 is compared, r1[*mk_e_^1^*] is only compared with r2[*mk_e_^1^*] and r1[*mk_e_^2^*] is only compared with r2[*mk_e_^2^*], and so on. This is because the match-key values are stored in the same order for all records.

This ordering of match-key values allows the calculation of frequency distributions of each match-key in the attack. An adversary can safely assume that a single column in the encoded database **D** (a column H_*j_ ∈ **H**) corresponds to a single encoded match-key. To prevent such assumptions by the adversary we propose to store match-key values in sets as opposed to lists. Each row **H**_i*_ ∈ **H** in the encoded database should be a set of match-key values where there is no order. Now the adversary cannot assume a single column represents a certain encoded match-key.

As opposed to improved privacy, there is a trade-off with this technique. A single match-key value from a record now needs to be compared with every match-key value from another record which will lead to increased time consumption. Furthermore, the possibility of obtaining false positives will also increase. For instance if two record r1 and r2 have the following attribute values: r1: *FirstName* = “Paul”, *LastName* = “Thomas”, *BirthYear* = “1992” and r2: *FirstName* = “Thomas”, *LastName* = “Paul”, *BirthYear* = “1992”, then match-key (F+B) of r1 and match-key (L+B) of r2 will have the same value and the record pair (r1, r2) will be classified as a match. Increasing false positives will reduce the overall quality of the linkage. However, this can be solved by prefixing an attribute specific value (such as an attribute qualifier or an attribute index) to the plain-text value before hashing. For example if we use ‘F’ for first name and ‘L’ for last name, we obtain match-key values such as “F-Thomas1992” and “L-Thomas1992”. This approach is different from record specific salting values as used in the context of Bloom filter encoding to avoid frequency attacks. Therefore, adding an attribute specific string however may not affect the frequency distribution, rather it is recommended to avoid the false positive rate.

**Recommendation 2:** As discussed in the Methods section, our proposed attack compares the correlations between the frequency distributions of encoded match-keys with plain-text match-keys. In order for this to be successful, the encoded match-keys need to have distinct frequencies for different match-key values. If the frequency distributions are close to uniform, an adversary will not be able to compare those distributions with plain-text frequency distributions.

As our second recommendation, we propose making the frequency distributions of encoded match-keys close or equal to uniform. In the encoded database **H**, we set the match-key values that have frequency larger than *x*, where *x* ≥ 1, as missing. This will ensure that all the remaining match-key values (hash-codes) for each encoded match-key are unique and the frequencies of all those match-key values will be between 1 and *x* making the distribution close to uniform. Hence, the adversary can no longer conduct any correlation analysis on these distributions since they are uniform and cannot be distinguished from each other. As with our first recommendation, this method also has a trade-off for improved privacy at the cost of reduced linkage quality. Setting match-key values as missing will lower the overall recall of the linkage since potential matches could be missed. At the same time, overall precision of the linkage will be improved since potential false positives could be removed when using this technique.

We conducted multiple experiments with *x* = 50, 20, 10, 5, 2, 1 where we set the match-key values with frequency > *x* as missing. As expected, the precision of the linkage has increased with a minimum and a maximum increase of +0.69% and +10.4%, respectively. The recall has dropped due to the true matches being missed because their match-key values are removed. The overall recall had a minimum and a maximum decrease of -0.04% and -1.29% respectively. The small changes in both precision and recall is because the number of records with match-key value frequencies > 1 is small compared to the 7 million records in the databases. However, if the databases have more errors or missing values in attributes this will potentially lead to a selection of encoded match-keys, H_*j_, with a small number of attributes which will improve the recall of the linkage. As we further discuss below, selecting encoded match-keys with a smaller number of attribute values (such as only 2 or 3 attributes in a H_*j_) will increase the frequencies of those encoded match-keys. In such a case using this recommendation to improve privacy will also lead to a potentially substantial reduction of recall.

It is possible to apply one or both of the above two recommendations to increase the privacy of the sensitive values in the encoded database. However, we recommend to initially apply the first recommendation and then check if any distinct frequency distributions can still be obtained and aligned between the plain-text and encoded databases. Depending on the results of the first modification we can then apply the second recommendation if further improvement of privacy is required. Furthermore, it is also worth noting that if the second recommendation is applied first, then there is no practical advantage of applying the first recommendation next because the attack will not be possible after the application of the second recommendation.

### Practical considerations of the multiple match-key encoding

While the original multiple dynamic match-key encoding proposed by Randall et al. [5] provides high linkage quality with acceptable privacy, there are few practical aspects that need to be taken into consideration when using this encoding for PPRL in real-world applications.

1. The selection of match-keys depends on the Fellegi and Sunter agreement and disagreement weights of individual attributes. In [5] the authors have conducted their experiments on selected match-key that provided the highest F-measure values for a given database. However, in practice these agreement and disagreement weights need to be estimated based on partially available ground truth or domain knowledge. Hence, with estimated values, there is no guarantee that the F-measure and thus linkage quality of the final record linkage will be optimal.
2. The multiple match-key encoding approach depends on exact matching of hash-codes. As also discussed by the authors in [5] this could be a disadvantage if the matching databases contain errors or variations in attribute values. Even a small typographical error or a character change (Christine vs. Christina) in an attribute value will lead to a completely different hash-code, whereas with approximate matching techniques such as Bloom filter encoding [15] this might not lead to false negatives. Furthermore, in our experiments, we observed that selected match-keys always contain a name attribute (*FirstName, MiddleName*, or *LastName*), as can be seen in Tables 4 and 5. This is because name attributes generally have more discriminatory power compared to other attributes used. Therefore, if the name attributes in the databases to be linked contain more errors or changes in their values, then this could lead to lower linkage quality despite low amounts of errors in other used attributes.
3. When the sizes of the databases get bigger, it is more likely for them to have a distinct frequency distribution for each match-key (attribute combination). Even if the authors of the multiple match-key encoding [5] suggested to have two or more attribute values in a match-key, our experiments showed that having even three attribute values in a match-key will still lead to a discrete frequency distribution for that particular match-key which can lead to re-identifications. In our experiments on databases with seven million records, the frequencies start to get close to uniform with match-keys with four or more attributes. But then again, if we are using five or more attributes for a match-key we need to make sure the attributes do not contain a large number of errors or missing values because that will lead to lower linkage quality.

## Conclusion

We have presented a privacy attack on the recently proposed multiple dynamic match-key encoding method for PPRL [5]. The proposed attack employs correlation calculations on frequency distributions to identify attribute combinations used to generate match-key values. Based on the identified match-keys, the attack then re-identifies attribute values that corresponds to individual match-key values (hash-codes). The experimental results showed that multiple dynamic match-key encoding is susceptible to frequency attacks under certain parameter settings. As countermeasures, we have proposed two recommendations to improve the privacy of the multiple match-key encoding approach and have discussed the trade-offs between linkage quality and improved privacy when using these methods. These recommendations show that with a small number of errors and missing values in databases, privacy can be strengthened while keeping high linkage quality.

As future work, we plan to investigate methods of improving the performance and the accuracy of the attack by analysing potential filtering techniques for match-keys. One such technique could be to analyse if multiple match-keys are the same for a given record pair. Such patterns of same match-keys in record pairs can potentially lead to the identification of attribute values used in encoded match-keys and thereby the re-identification of encoded plain-text values.

## Acknowledgments

This work was funded by the Australian Research Council under DP160101934.

## Ethics statement

No ethics approval was required for this study, because all the databases used in this study are publicly available and necessary references are provided for them.
